# No evidence of predicted phenotypic changes after hurricane disturbance in a shade-specialist Caribbean anole

**DOI:** 10.1098/rsbl.2022.0152

**Published:** 2022-08-03

**Authors:** Miguel A. Acevedo, David Clark, Carly Fankhauser, John Michael Toohey

**Affiliations:** ^1^ Department of Wildlife Ecology and Conservation, University of Florida, Gainesville, FL 32611-7011, USA; ^2^ Department of Biological Sciences, University of Pittsburgh, Pittsburgh, PA 15261, USA

**Keywords:** extreme climatic events, hurricanes, Puerto Rico, limb length, disturbance, adaptation

## Abstract

Extreme climatic events (ECEs) such as hurricanes have been hypothesized to be a major driving force of natural selection. Recent studies argue that, following strong hurricane disturbance, *Anolis* lizards in the Caribbean undergo selection for traits such as longer forelimbs or smaller body sizes that improve their clinging ability to their substrates increasing their chances of surviving hurricane wind gusts. Some authors challenge the generalization of this hypothesis arguing that other mechanisms may explain these phenotypic changes or that they may not necessarily be generalizable across systems. To address this issue, we compared body size and relative forelimb length of *Anolis gundlachi*, a trunk–ground anole living in closed-canopy forests in Puerto Rico, before, four months after, and 15 months after Hurricanes Irma and Maria in 2017. Overall, our results show no clear evidence of a temporal decrease in body size or increase forelimb length (relative to body size) challenging the generalizability of the clinging ability hypothesis. Understanding how animals adapt to ECE is an emerging field. Still, we are quickly learning that this process is complex and nuanced.

## 1. Introduction

Extreme climatic events (ECEs) are rare occurrences that drive ecosystems away from their levels of natural variability and are expected to become more common with increasing climate change [1,2]. When populations undergo ECE, theory predicts various potential outcomes. On one extreme, populations can go locally extinct if the population cannot recover from high mortality events (e.g. [3,4]). Alternatively, trait phenotypes can change through a variety of mechanisms including plasticity or evolutionary change (e.g. [5]). Supporting evidence for these predictions is scarce, in part, because ECEs are rare and stochastic, which hinders the possibility of appropriate experimental design. Still, serendipitous opportunities sometimes allow for the collection of baseline data before an ECE that can be leveraged for comparisons with conditions after the event [6]. This has been the *modus operandi* of studies aimed to understand the eco-evolutionary consequences of tropical storms and hurricanes [7].

Hurricanes are catastrophic stochastic events that drive changes to natural systems at multiple scales [8]. These can include changes in soil nutrient composition (e.g. [9]), shifts in species interactions (e.g. [10]), phenotypic changes (e.g. [11]) and severe mortality events that result in short-term declines in population densities (e.g. [12]). Still, there is mounting evidence in plant systems that hurricane-prone regions are also highly resilient to disturbance (e.g. [13]). Still, we know less about the potential role that hurricane disturbance plays in the selection of phenotypic traits in animals.

Recent studies on *Anolis* lizards following landfall of Hurricanes Irma and Maria in the Caribbean in 2017 suggest that hurricane disturbance selects for clinging ability via related phenotypic traits such as smaller body sizes, longer forelimbs and larger toepads [11,14,15]. These studies hypothesize that lizards with these traits have a better ability to hang to their substrates and not succumb to hurricane wind gusts. These traits are hypothesized to be inherited by subsequent generations after the selective survival of individuals with better clinging ability. The clinging ability hypothesis has been key to interpret the higher prevalence of larger toepads in hurricane-prone regions [16]. Still, this pattern is not necessarily unequivocal with studies finding lack of evidence for this pattern in other systems [17]. Moreover, even in cases when longer limbs are selected after the hurricane, some challenge the generalizability of the clinging mechanism as an ultimate explanation where locomotor performance, availability of retreat areas and changes in vegetation structure may ultimately lead to similar phenotypic patterns [18]. Limb length in anoles is a plastic trait that can also evolve responding to multiple selective pressures including substrate size [19]. Therefore, in the aftermath of a hurricane, multiple evolutionary pressures of varying strengths may act simultaneously resulting in alternative system-specific temporal phenotypic variation patterns. Additional studies quantifying phenotypic traits before and after major hurricane disturbances are needed to assess if the hypothesized pattern is generalizable.

Here, we quantified body size and relative forelimbs length in *Anolis gundlachi*, a trunk–ground anole living in closed-canopy forests in Puerto Rico before, four months, and 15 months after Hurricanes Irma and Maria in 2017. Following the population for a period that spans more than 2 years allows insights into the hypothesis that selection for traits that improve clinging ability persist in the population shortly after the hurricane, and at least one generation after the disturbance. Supporting evidence for this hypothesis would include a decrease in the average body size, and a higher prevalence of individuals with longer forelimbs relative to their body size four months and 15 months after the storm. By contrast, the lack of evidence of these shifts would suggest that the storm was not a significant selection event for these traits.

## 2. Material and methods

### (a) Study system

We quantified phenotypic traits (i.e. body size and forelimb length) of the lizard *A. gundlachi* in 10–13 January 2017 (pre-hurricane), 12–16 January 2018 (four months after the hurricane) and in 16–22 December 2018 (15 months after the hurricane) at El Verde Field Station in Puerto Rico. Quantifying phenotypic traits consistently in the same season allowed us to control for unmeasured seasonal environmental factors and ontogenic variability. It is still possible that a small proportion of individuals persisted among sampling periods. Before Hurricanes Irma and Maria in 2017, the site had been relatively undisturbed by a major atmospheric event since Hurricane Georges in 1998. Therefore, the anole populations had experienced no major hurricane disturbance for around 19 generations before the 2017 hurricane season. In the 2017 season, Hurricane Maria was the strongest, producing winds of up to 250 km h^−1^ and 15% of the average yearly precipitation in just 24 h [20]. The centre of the hurricane was located around 28 km of our field site causing a 51% loss in greenness and greater than three times more stem breaks than previous storms in the latter twentieth century [21,22]. The large magnitude of the disturbance allows us to detect potential effects that may not be necessarily apparent from smaller disturbances.

### (b) Data collection

We quantified snout-to-vent length (SVL), and forelimb length on captured individuals of *A. gundlachi*—a medium-sized lizard member of the trunk–ground ecomorph—around the main trails of the LFDP (e.g. [23]; see electronic supplementary material, appendix 1 for more sampling details). We restricted our analyses to adult individuals (greater than 40 mm SVL). To address if potential phenotypic changes are related to shifts in the distribution of substrate sizes, we also measured the diameter at breast height (DBH) of the substrate where lizards were caught. Note that we did not collect substrate size data in the January 2018 sampling, but we collected similar data in a sampling during the summer of 2018 right before the hurricanes. Therefore, to compare substrate size, we used the DBH data of each tree from the summer of 2018 as our baseline category.

### (c) Statistical analyses

We followed a similar approach to Donihue *et al*. [14] for comparative purposes: we tested for changes in the log of body size (i.e. SVL), and log of forelimb length (radius/ulna and humerus) using three separate linear models that predicted each phenotypic trait as a function of sampling time (before, four months, or 15 months after hurricane disturbance), with sex (males or females) as a controlling variable. The log transformation of the response variables aids meeting the assumption of normality of the residuals. The models used to test for changes on forelimb length also had the log of SVL as an additional controlling variable. In the analyses of forelimb length, we also added a random effect of sampling date within sampling time to account for within observer measurement variability and within sampling season among-trail variability. We used a likelihood ratio test to assess if the random effects improved model fit. To make inferences about the role of sampling time on changes in phenotypic characteristics, we compared a model with and without sampling time using the Kenward–Roger conditional *F* test (electronic supplementary material, appendix 1).

We conducted an additional *post hoc* analysis to assess if differences in phenotypic traits could be attributed to changes in substrate size by modelling the DBH of tree and palm substrates where the lizards were caught as a function of sampling time with sex as a controlling variable using a linear model.

## 3. Results

**Figure 1.**
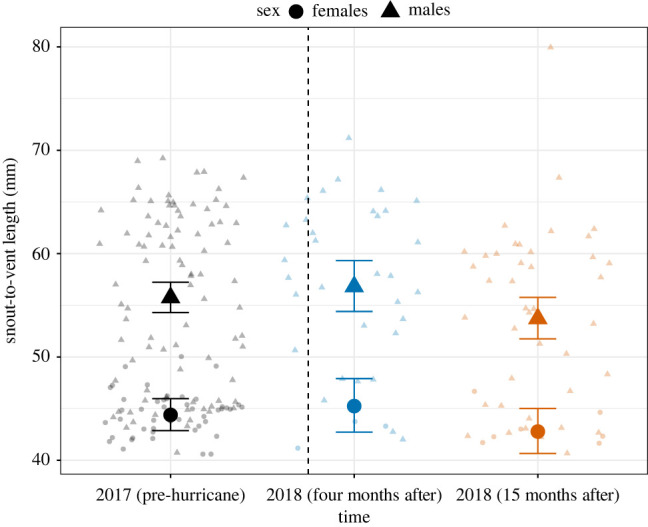
The figure shows the distribution of body sizes of males and females *A. gundlachi* at El Verde Field Station in Puerto Rico before, four months, and 15 months after Hurricanes Irma and Maria in 2017. The large symbols and error bars represent mean and 95% confidence intervals predictions from the linear model. The smaller symbols in the background represent the data points. The dotted line represents the relative time of the disturbances. Colours represent the different sampling periods. These are consistent among all figures. The analyses show no significant difference in SVL in this period.

To assess potential changes in phenotypic characteristics, we analysed a total of *N* = 216 individuals across the three sampling times (electronic supplementary material, appendix 1). We found no clear statistical difference between the body size of individuals four months (*β* = 0.01 ± 0.03 s.e., *p* = 0.46) or 15 months after the hurricane (*β* = −0.04 ± 0.02 s.e., *p* = 0.10) and the size of individuals before the hurricanes (*R*^2^ = 0.38; figure 1). As expected, the model predicted that males are larger than females (males: *β* = 0.23 ± 0.02 s.e., *p* < 0.01; electronic supplementary material, table S1).

**Figure 2.**
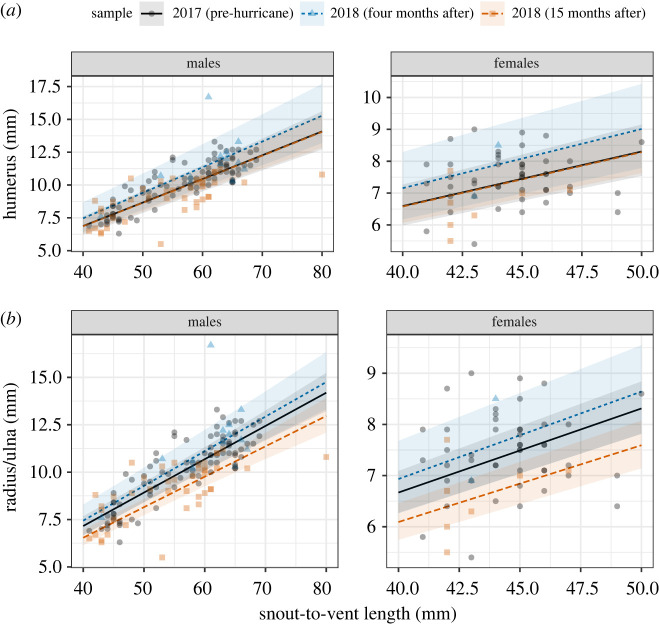
The figure shows model predictions for the relationship between limb length and body size for male and female *A. gundlachi* before, four months and 15 months after Hurricanes Irma and Maria. The lines represent the model's best fit and shaded areas 95% confidence intervals.

We also did not find clear statistical evidence of temporal changes in the relative length of the humerus (*F_kr_* = 0.55, *p* = 0.60; *F_pb_* = 1.08, *p* = 0.66). Specifically, the model predicted no clear difference between the relative length of the humerus before and four months (*β* = 0.08 ± 0.09 s.e.; figure 2*a*) or 15 months after the hurricanes (*β* = −0.0025 ± 0.06 s.e.; electronic supplementary material, table S2). A likelihood ratio test comparing a model with and without the within-season sampling date as a random effect shows strong support for the incorporation of these random effects (LRT = 20.59, *p* < 0.001).

Overall, we found no clear statistical evidence of changes across time in the relative length of the radius/ulna (*F_kr_* = 4.10, *p* = 0.05; *F_pb_* = 7.63, *p* = 0.06). The model predicted no clear difference between the relative length of the radius/ulna before and four months after the hurricanes (*β* = 0.04 ± 0.06 s.e., electronic supplementary material, table S3). However, the model predicted, with less uncertainty, that relative limb length 15 months after the hurricane were 0.91 times shorter than those before the disturbance (*β* = −0.09 ± 0.04 s.e.; figure 2*b*). A likelihood ratio test comparing a model with and without the within-season sampling date as a random effect shows strong support for the incorporation of these random effects (LRT = 6.14, *p* = 0.001).

The slight decrease in radius/ulna relative length 15 months after the hurricane is not necessarily explained by lizards using smaller substrates. We found no clear statistical difference between the size of substrates used before and four months after the hurricanes (*β* = 1.63 ± 1.42 s.e., *p* = 0.253; electronic supplementary material, table S4). In fact, 15 months after the disturbances lizards were using substrates that were on average 3.24 cm larger in diameter compared to pre-hurricane conditions (1.24 s.e., *p* = 0.01; electronic supplementary material, figure S1).

## 4. Discussion

Hurricanes are an example of ECE that are predicted to increase in intensity with climate change [24]. Previous studies argue that severe hurricane disturbance results in selection for longer forelimbs and smaller body sizes in anoles because these traits increase their clinging ability [14]. Our analyses in a shade-specialist in Puerto Rico do not support this hypothesis. Similarly, a study quantifying traits of *A. cristatellus*—an open/disturbed area specialist*—*before and after hurricane disturbance also found no support for phenotypic changes that favour clinging ability [17]. What can explain this contrasting result on studies following the same hurricane disturbances in different islands?

Empirical support for the clinging ability hypothesis comes from anoles living in sandy coastal habitats in smaller islands (less than 3 km^2^) such as *Anolis scriptus* in Pine Cay and Water Cay, in the Bahamas and *A. carolinensis* on SL9 and SL10 islands in the Indian River Lagoon in Florida [11,14]. Caribbean tropical moist forests have larger trees than coastal habitats. Therefore, morphological traits that improve clinging ability may be more important in coastal habitats where the lizards can wrap their limbs around smaller substrates. Also, the selection pressure caused by high-speed hurricane winds can be higher in these smaller islands where wind gusts can expel lizards to the ocean where they have slim chances of survival. By contrast, the selection pressure for these traits in Puerto Rico may be lower. Our study species, *A. gundlachi*, perches mostly in trees and palms in tropical moist forest (greater than 110 km^2^) at least 10 km from the coast. Therefore, individuals that survive being expelled from their trees, may find refuge on the forest understory increasing their likelihood of survival.

While we found, overall, no clear evidence supporting the clinging ability hypothesis, our models predicted a slight decrease in the relative length of the radius/ulna 15 months after the disturbances. Limb length in anoles often correlates with substrate size as a result of plasticity or adaptation (e.g. [19,25]). Therefore, a plausible hypothesis would be that a decrease in limb length could be a phenotypic response to a decrease in the average size of available substrates due to high mortality of larger trees following the hurricanes [22]. However, we found that the average diameter of substrates used slightly increased in the same period (electronic supplementary material, figure S1) suggesting that, if adaptive, this decrease in relative limb length may be driven by other mechanisms. Smaller relative forelimbs could be the result of increasing temperatures driving developmental changes. Increasing temperatures is likely an important selection force for *A. gundlachi* because it is a thermoconformer particularly sensitive to increases in temperature [26]. Hurricanes cause high defoliation of the forest canopy resulting in a marked increase in temperature [27]. Variation in limb length in many species of anoles occurs early in development [28], and previous studies show that increasing temperature can decrease embryonic survival and developmental retardation [29]. While we found no evidence of the predicted changes in forelimb length and body size, we did not have data on toepad size, which was another trait key in previous studies. Therefore, the potential for toepad size to follow the predictions of the hypothesis remains to be tested. Nevertheless, toepad size in *A. cristatelus* in forest habitats in Puerto Rico decreased four months after the hurricane, a pattern contrary to that predicted by the hypothesis [17].

The clinging ability hypothesis is mechanistically plausible and empirically supported. However, our results combined with other recent studies showing lack of support in different systems suggest that there may not be enough evidence to suggest the hypothesis is generalizable. Patterns of function-mediated selection on morphological traits vary with form–function relationships changing across environments. Moreover, multiple alternative evolutionary forces may interact in complex ways after hurricanes and other ECE. Understanding how animals adapt to ECE is an emerging field. Still, we are quickly learning that this process is complex and nuanced.

## Data Availability

Data are available from the Dryad Digital Repository at https://doi.org/10.5061/dryad.fxpnvx0tz [30] and all code for statistical analyses is available at https://github.com/maacevedo.
